# Light-Induced Surface Tension Gradients for Hierarchical Assembly of Particles from Liquid Metals

**DOI:** 10.1021/acsami.2c20116

**Published:** 2023-02-02

**Authors:** Jiayun Liang, Zakaria Y. Al Balushi

**Affiliations:** † Department of Materials Science and Engineering, University of California, Berkeley, Berkeley, California94720, United States; ‡ Materials Sciences Division, Lawrence Berkeley National Laboratory, Berkeley, California94720, United States

**Keywords:** Laguerre-Gaussian lasers, Marangoni effect, liquid-gallium, nanoparticles, assembly

## Abstract

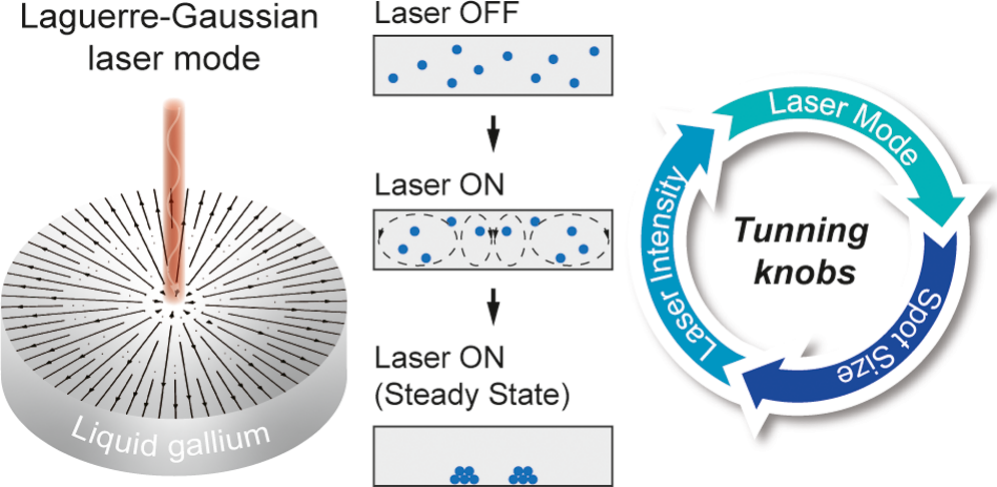

Achieving control over the motion of dissolved particles in liquid metals is of importance for the meticulous realization of hierarchical particle assemblies in a variety of nanofabrication processes. Brownian forces can impede the motion of such particles, impacting the degree of perfection that can be realized in assembled structures. Here, we show that light-induced Marangoni flow in liquid metals (i.e., liquid-gallium) with Laguerre-Gaussian (LG_pl_) lasers as heating sources is an effective approach to overcome Brownian forces on particles, giving rise to predictable assemblies with a high degree of order. We show that by carefully engineering surface tension gradients in liquid-gallium using non-Gaussian LG_pl_ lasers, the Marangoni and convective flow that develops in the fluid drives the trajectory of randomly dispersed particles to assemble into 100 μm wide ring-shaped particle assemblies. Careful control over the parameters of the LG_pl_ laser (i.e., laser mode, spot size, and intensity of the electric field) can tune the temperature and fluid dynamics of the liquid-gallium as well as the balance of forces on the particle. This in turn can tune the structure of the ring-shaped particle assembly with a high degree of fidelity. The use of light to control the motion of particles in liquid metals represents a tunable and rapidly reconfigurable approach to spatially design surface tension gradients in fluids for more complex assembly of particles and small-scale solutes. This work can be extended to a variety of liquid metals, complementary to what has been realized in particle assembly out of ferrofluids using magnetic fields.

## Introduction

Achieving control over the motion of particles in fluids is important to realize the high degree of perfection needed in the assembly processes of nano-to-macroscale objects.^1−4^ An important force that must be taken into consideration in the assembly of such particles is Brownian motion.^5−7^ One approach to reduce the impact of Brownian on particles dispersed in liquids is by taking advantage of the Marangoni effect.^8,9^ Marangoni effect (i.e., Marangoni flow) describes fluid flow induced by surface tension gradients. Liquids with higher local surface tension pull strongly around the surrounding. The resulting shear stress at the two-phase interface gives rise to a rather strong convective motion of the fluid, naturally flowing from regions of low local surface tension to high ones. This surface tension gradient in fluids can be induced by several means, for example, by adding surface surfactants,^10,11^ tailoring the solute concentration,^12^ or developing a temperature gradient on the surface using a variety of heating sources.^13−15^ Much of the research in this field has been focused on controlling Marangoni flow in transparent hemispherical droplets. In this case, fluid flows away from the apex of the droplet to compensate for the liquid loss at the edges. Particles dispersed in these droplets end up accumulating at the edges, giving rise to the seminal “coffee-ring” effect.^16^ By inducing Marangoni flow in the opposite direction, one can suppress the coffee-ring effect and achieve a more uniform particle pattern,^10^ or even the “reverse coffee-ring” effect,^17−19^ where particles accumulate at the center of the droplet upon evaporation. Furthermore, the Marangoni effect has also been observed in opaque liquids, like liquid metals, such as in a variety of crystal growth directional solidification processes.^20−23^ Such liquid metals have also been used as catalysts during the fabrication of thin films and nanostructures by the vapor–liquid–solid (VLS^24−28^), liquid–liquid–solid (LLS^29,30^), and solid–liquid–solid (SLS^31^) growth mechanisms of materials. The limitation of these crystal growth processes was that the size and geometry of the precipitating crystalline solid out of the liquid metal were always confined by the geometry of the liquid metal itself.^28,32−34^ Therefore, if one could control the fluid flow of opaque liquids and therefore the motion of small-scale solutes during the fabrication of materials or assemblies out of liquid metals, it could be possible to create material designs out of the liquid metal with varying degrees of complexity.

Herein, we model the light–matter interaction of laser heating sources to spatially structure surface tension gradients on liquid metals to therefore control the motion and assembly of particles *via* the Marangoni effect. In this work, Laguerre-Gaussian (LG_pl_) lasers were utilized to engineer surface tension gradients and induced Marangoni flow. Here, *p* and *l* are radial and azimuthal index numbers for the LG_pl_ laser, respectively. The LG_pl_ lasers were selected because compared to a Gaussian laser, they operate in higher-order transverse modes, which determines the intensity distribution in the cross section of the laser beam, where higher-order transverse modes lead to a higher complexity in this intensity distribution. Therefore, LG_pl_ lasers allow for more degrees of freedom in the design of the temperature gradient and, thus, Marangoni flow patterns. In this work, liquid-gallium was used as a model liquid metal because of its low melting point, high boiling point, and relatively low vapor pressure over a wide temperature range.^35^ Furthermore, liquid-gallium does not form compounds with many elements (e.g., tungsten, silicon, germanium, carbon, etc., Table S1) and is therefore useful in a variety of crystal growth processes.^25,34,36,37^ In this work, a ring pattern assembly of tungsten particles out of liquid-gallium was realized by strategically choosing the radial and azimuthal index numbers as well as beam conditions for the LG_pl_ laser interacting with the surface of liquid-gallium. The assembled ring-shaped particle patterns were preferentially formed because of unique fluid flow vortices that form within the bulk of liquid-gallium, essentially creating a “forbidden zone” of assembly. This allows one to specifically structure assembled particle features between the coffee-ring and reverse coffee-ring effects. The perfection of the assembly was quantified by assessing the entropy of these patterns, which improved with the existence of significant drag forces on the particles that could overcome the Brownian motion of the particles due to Marangoni flow.

## Results and Discussion

**Figure 1:**
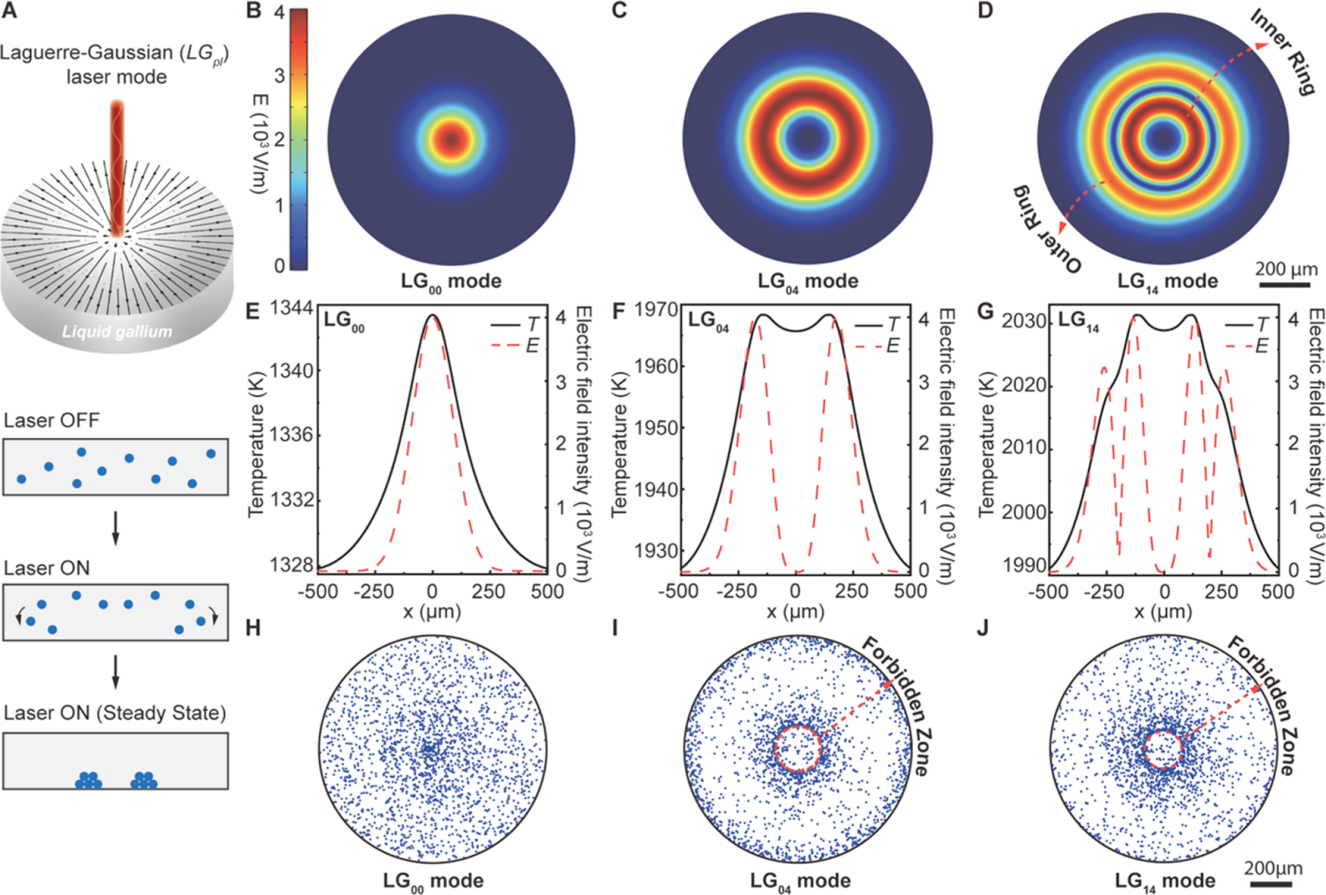
Electric field profiles, temperature profiles, and resulting particle patterns assembled out of liquid-gallium under different Laguerre-Gaussian (LG_pl_) laser modes. (A) Schematic highlighting the process of assembling particles out of liquid-gallium using a LG_pl_ lasers (laser wavelength, λ = 645 nm and spot size, *w*_o_ = 125 μm) When LG_pl_ lasers interact with liquid-gallium, Marangoni flow develops, guiding randomly dispersed tungsten particles (diameter, *d*_p_ = 20 μm) to assemble at the liquid–solid interface. (B–D) Profile of the electric field on the surface of liquid-gallium under (B) LG_00_, (C) LG_04_, and (D) LG_14_ lasers, respectively. An inner and outer ring are observed in the doughnut-shaped electric field profile for (D) LG_14_ laser, as highlighted by the two dashed red arrows. The scale bar for all of the electric field profiles is 200 μm. (E–G) Profiles of the electric field (dashed red lines) and temperature (solid black lines) on the surface of liquid-gallium. (H–J) Resulting particle patterns at the liquid–solid interface for (H) LG_00_, (I) LG_04_, and (J) LG_14_ lasers interacting with liquid-gallium. The ring-shaped particle patterns are obtained with (I) LG_04_ and (J) LG_14_ laser modes only, creating a forbidden zone of assembly, where tungsten particles do not gather, which is highlighted by dashed red lines. The scale bar for all particle patterns is 200 μm.

Laser heating is useful in engineering fluid flow.^38,39^ Lasers can also be tuned through a variety of parameters, including wavelength (λ), spot size (*w*_o_), and the profile of the electric field. This makes lasers highly accessible as rapid and reconfigurable heating sources for engineering temperature gradients in fluids. So far, Gaussian lasers have only been applied as a heating source to induce Marangoni flow at liquid–solid and liquid–liquid interfaces of transparent liquids.^40^ The use of non-Gaussian lasers to engineer more complex temperature gradients in transparent or opaque liquids has been largely unexplored. Therefore, to illustrate the uniqueness of using a laser to structure the surface tension gradient and therefore convective flow patterns in liquid metals, the interaction of LG_pl_ lasers (λ = 64*5* nm and *w*_o_ = 125 μm, Figure 1) onto the surface of liquid-gallium with 2000 randomly dispersed tungsten particles with a diameter (*d*_p_) of 20 μm was investigated. As illustrated in Figure 1A, the interaction of the laser will induce Marangoni flow for the hierarchical assembly of these randomly dispersed particles at the liquid–solid interface.

To reveal the relationship between the LG_pl_ laser and the Marangoni flow, three different LG_pl_ lasers (i.e., LG_00_, LG_04_, and LG_14_, see the Methods Section) were investigated. The electric field distribution (*E*) on the surface of the liquid-gallium is shown in Figure 1B–D. Unlike the Gaussian distribution of the electric field with the LG_00_ laser (Figure 1B), the electric field distribution of the LG_04_ and LG_14_ lasers was doughnut-shaped (Figure 1C,D, respectively). That is, the peak intensity of the electric field was not at the center of the surface of liquid-gallium (Figure 1E–G, dashed red lines). Unlike the single electric field ring maximum in the LG_04_ laser seen in Figure 1C, the LG_14_ laser contained two ring maxima in the electric field distribution over the surface of liquid-gallium. These rings are labeled as “inner” and “outer” rings in Figure 1D (dashed red arrows). The interaction of these lasers with liquid-gallium leads to electromagnetic heating (*Q*_e_) of the surface, which is given by
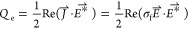
where σ_f_ is the electrical conductivity and *J* is the current density of liquid-gallium. The surface temperature profiles of liquid-gallium due to the interaction of these three LG_pl_ lasers are also illustrated in Figure 1E–G. Since the intensity of the electromagnetic heating was proportional to the square of the strength of the electric field, the surface temperature profiles of liquid-gallium were composed of single (Figure 1E), double (Figure 1F), and quad (Figure 1G) temperature maxima for the LG_00_, LG_04_, and LG_14_ lasers, respectively. Though the maximum intensity values of the electric field were equal for the LG_00_, LG_04_, and LG_14_ lasers (4 × 10^3^ V/m), due to the variation in the electric field distribution between these three lasers, the amount of electromagnetic heating and therefore resulting temperature maxima of liquid-gallium using each laser differed. This strong dependence of the electromagnetic heating of liquid-gallium to the electric field of the laser highlights the ability to engineer a variety of unique temperature gradients in liquid-gallium with a high degree of versatility.

Moreover, the temperature gradient that develops on the surface of liquid-gallium induces sheer stresses at the gas–liquid interface, giving rise to Marangoni flow
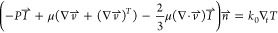
where *v⃗* is the velocity of the fluid and *k*_0_ is the temperature coefficient of the surface tension.^41^ Over time, the randomly dispersed tungsten particles pick up drag forces and follow the convective flow streamlines within the bulk of the liquid-gallium. Finally, these particles assembled at the liquid–solid interface into a variety of patterns, which depended on the conditions of the LG_pl_ laser. The resulting particle patterns are highlighted in Figure 1H–J. Figure 1H shows randomly distributed tungsten particles across the solid surface when the LG_00_ laser was used as a heating source. However, when the LG_04_ and LG_14_ lasers were utilized, ring-shaped particle patterns were assembled out of the liquid–solid interface, as shown in Figure 1I,J for the LG_04_ and LG_14_ lasers, respectively. The use of the LG_04_ and LG_14_ lasers essentially created regions where particles could not assemble on the solid surface. These regions are referred to as the forbidden zone which form due to unique vortices that develop within the flow pattern in the bulk of liquid-gallium.

### Influence of Marangoni Flow on Brownian and Particle Assembly

**Figure 2:**
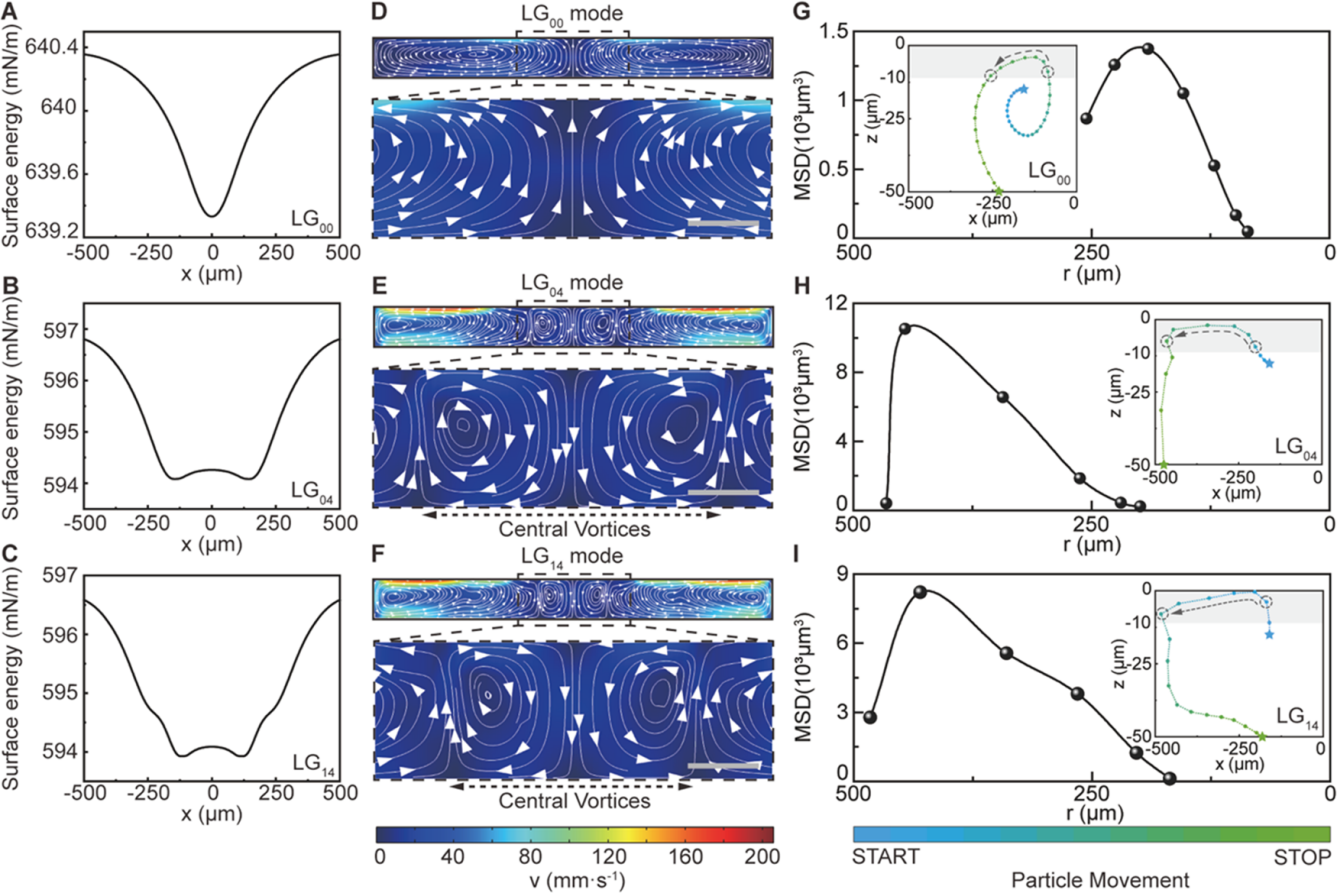
Influence of surface tension gradients of liquid-gallium on the particle motion in the bulk fluid. (A, B, and C) Surface tension profiles of liquid-gallium along the x-axis for (A) LG_00_, (B) LG_04_, and (C) LG_14_ lasers (λ = 645 nm and *w*_o_ = 125 μm). (D, E, and F) Convective flow patterns induced by the surface tension gradient (i.e., Marangoni flow) for (D) LG_00_, (E) LG_04_, and (F) LG_14_ lasers. For (E) LG_04_ and (F) LG_14_ lasers, two additional central vortices are observed in the convective flow patterns in the fluid, as highlighted by dashed black lines under the magnified regions (scale bars are 50 μm). The absolute value of the fluid velocity is indicated by the color bar at the bottom. (G, H, and I) Mean-squared displacement (MSD) versus radial position (*r*) of for particles under (G) LG_00_, (H) LG_04_, and (I) LG_14_ lasers when the particle moves near the top surface of liquid-gallium (|*z*| ≤ 10 μm, shaded gray background). The insets in panels (G, H, and I) are the particle trajectories (*blue-to-green gradient*) guided by the fluid flow induced under the (G) LG_00_, (H) LG_04_, and (I) LG_14_ laser modes, respectively. All particles were released at the same position in the fluid (blue star) and finally assembled at the liquid–solid interface (green star).

The pattern of the assembled particles observed in Figure 1H–J was highly dependent on the electric field distribution and the consequentially developed surface tension gradients when the LG_00_ (Figure 2A), LG_04_ (Figure 2B), and LG_14_ (Figure 2C) lasers interacted with the surface of liquid-gallium. To understand the origin of the forbidden zone, the Marangoni and resulting convective flow patterns within the depth of liquid-gallium, as a function of the laser, were investigated (Figure 2D–F). Fluid flow induced by the LG_00_ laser (Figure 2D) led to the formation of two vortices, allowing liquid-gallium to recirculate from the center to the periphery of the bulk. However, the formation of two additional vortices within the center of the convective flow pattern (i.e., “central vortices”) was prominent when the LG_04_ (Figure 2E) and LG_14_ (Figure 2F) lasers interacted with liquid-gallium. A substantial enhancement in the velocity of the fluid can also be seen in Figure 2E,F. This enhanced fluid velocity will strongly dictate the trajectory of the particles dispersed in the fluid. This is evident in Figure 2G–I, which highlights the mean-squared displacement (MSD, see the Supporting Information—Section II), for particles moving near the surface of liquid-gallium (|*z*| ≤ 10 μm) as a function of the LG_pl_ laser. The corresponding *xz* trajectories of the particles are also included as insets in Figure 2G–I. In the case of the LG_04_ (Figure 2H) and LG_14_ (Figure 2I) lasers, the peak of the MSD shifts further away from the center of liquid-gallium compared to the LG_00_ laser (Figure 2G). Since the values of the MSD reflects the dynamics of the surrounding fluid, the emergence of these new central vortices with outward flow will ultimately play a key role in preventing particles from remaining at the center of the fluid.

**Figure 3:**
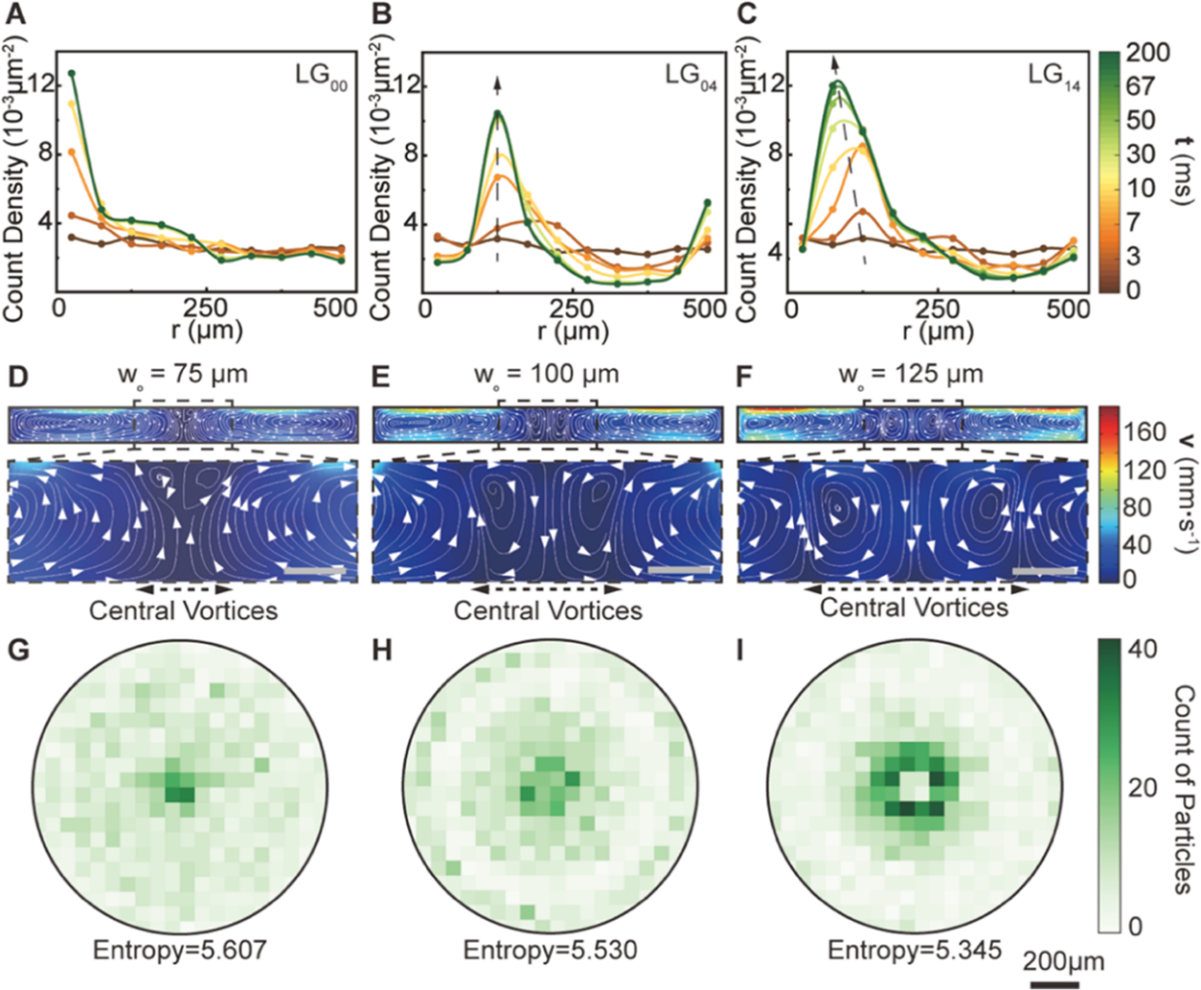
Impact of the laser mode and spot size on the particle pattern assembled out of liquid-gallium. (A–C) Time-dependent radial (*r*) particle count density for 2000 randomly released tungsten particles (*d*_p_ = 20 μm) in liquid-gallium guided by the convective flow induced by (A) LG_00_, (B) LG_04_, and (C) LG_14_ lasers. All 2000 particles were released at *t* = 0 *s*, and all settled at the liquid–solid interface at *t* = 0.2 s. The width of the radial bin for the particle count density profiles was set to 50 μm. (D–F) Fluid flow pattern in liquid-gallium under LG_14_ laser with the spot size of (A) 75 μm, (C) 100 μm, and (E) 125 μm. Two additional central vortices are magnified for clarity (scale bar is 50 μm). The absolute value of the fluid velocity is indicated by the color bar (red-to-blue gradient) on the right. (G–I) Density distribution maps (bin size = 50 μm) and corresponding entropy values for particle patterns induced by fluid flow under the LG_14_ laser with a spot size (*w*_o_) of (G) 75 μm, (H) 100 μm, and (I) 125 μm. The scale bar for all density distribution maps is 200 μm.

The spatial extent of the “central vortices,” and therefore the forbidden zone, was smaller in the LG_14_ laser compared to that in the LG_04_ laser. This was due to the existence of the inner and “outer” rings in the electric field distribution, as alluded to in Figure 1D. More broadly, the influence of the central vortices on the spatial extent of the forbidden zone can be further supported by assessing the time dependence of the radial (*r*) particle count density of 2000 randomly released tungsten particles (*d*_p_ = 20 μm, Figure 3A–C). When the particles were initially released (*t* = 0s), the particle count density across the radial direction of the liquid–solid interface were very close to each other, oscillating around 3.15 × 10^–3^ μm^–2^ with a mean-squared error of 0.27 × 10^–3^ μm^–2^. Over time, the particle count density at *r* ≤ 50 μm in LG_00_ increased (Figure 3A), while in LG_04_ and LG_14_, it decreased (Figure 3B,C). At *t* = 0.2 s, the particle count density for the LG_14_ laser around the central region of the liquid–solid interface was 2.55 × 10^–3^ μm^–2^ (Figure 3C, solid green line), which was an 80% reduction in the particle count density when compared to the LG_00_ laser (12.73 × 10^–3^ μm^–2^, Figure 3A, solid green line). Also, as time increased, the peaks in the particle count density also increased (Figure 3B,C, dashed black arrows). The peak positions for the LG_04_ and LG_14_ lasers were at *r* = 125 and 75 μm from the center of the liquid–solid interface, respectively. This elucidates why a smaller diameter in the forbidden zone was observed in the LG_14_ laser when compared to LG_04_ laser (Figure 1I,J, respectively). Therefore, the existence of ring features in the electric field distribution in the LG_pl_ laser allows one to spatially control the extent of the central vortices, and controlling other parameters of the laser beam itself could further tune the extent of the “forbidden zone.”

Moreover, the significance of the central vortices on particle assembly at the liquid–solid interface was further corroborated by investigating the effect of the LG_14_ laser spot size (*w*_*o*_) on fluid flow. Evident in Figure 3D was the fluid flow that results from the interaction of a 75 μm LG_14_ laser with liquid-gallium. At this spot size, the spatial extent of the central vortices in the fluid was rather negligible. However, as the *w*_o_ increased to 100 μm (Figure 3E) and 125 μm (Figure 3F), the appearance of the “central vortices” was substantial, which increased in extent with increasing *w*_o_. The absence of a fully developed central vortex in Figure 3D allowed for particles to freely circulate at the center of the liquid. In this case, particles that would assemble out of the liquid–solid interface would have a lower degree of order. To highlight this, we introduce the concept of entropy (see the Supporting Information—Section III) to quantify this degree of order from the particle density maps as a function of *w*_o_ (Figure 3G–I). A decrease in the value of entropy alludes to a higher degree of order in the particle distribution maps. As the *w*_o_ increased from 75 to 100 μm and to 125 μm, the value of entropy was reduced from 5.607 to 5.530 and 5.345, respectively. This means that the 125 μm *w*_o_ for the LG_14_ laser had the highest degree of order, as reflected in the well-defined ring feature of the assembled particles seen in the distribution map in Figure 3F. Interestingly, it can be observed that 54.2% of the particles preferred to gather around the forbidden zone of the solid surface. This was because as the *w*_o_ increased, the fully developed central vortices repelled particles from entering the central region of the fluid. This shows that the drag forces on the particle can significantly overcome Brownian forces to assemble patterns with a high degree of order.

### Investigating the Interacting Forces Impacting Particle Assembly

To gain a deeper understanding on how particles gather around the periphery of the forbidden zone when the LG_14_ laser (*w*_*o*_ = 125 μm) was utilized, the interacting forces on the moving particles in the fluid were investigated. Evidently, the synergy between the Marangoni flow and the effective gravitational forces on the particle was responsible in driving the formation of the ring-shaped patterns. This effective gravitational force (*G*_eff_) *is g*iven by
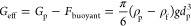
where *G*_p_, *F*_buoyant_, and *d*_p_ are the gravitational force, the buoyant force, and the diameter of the particle, respectively. Since the density of the tungsten particle (ρ_p_) was higher than that of the fluid (ρ_f_), the direction of the *G*_eff_ points vertically downward. The drag force (*F*_D_) from the Marangoni flow and/or convective flow around the particle is given by
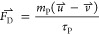
where *u⃗* is the velocity of the particle and *v⃗* is the velocity of fluid flow. The characteristic time for the particle (τ_*P*_) is expressed by
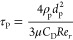
where μ is the dynamic viscosity of liquid gallium and *Re*_*r*_ is the relative Reynolds number of particles in the flow. *C*_*D*_ is the drag coefficient coupling the velocity of a particle to the surrounding fluid velocity. Relative Reynolds number (*Re_r_)* is calculated with
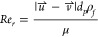
And the relationship between *C*_D_ and *Re_r_* is^42^
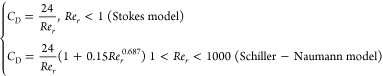
Considering the Re_r_ is less than 1 for most of the time steps in our simulations, if not otherwise specified, results shown in this work are simulated with drag coefficient derived from Stokes law (*C*_D_ = 24/*Re_r_*).

**Figure 4:**
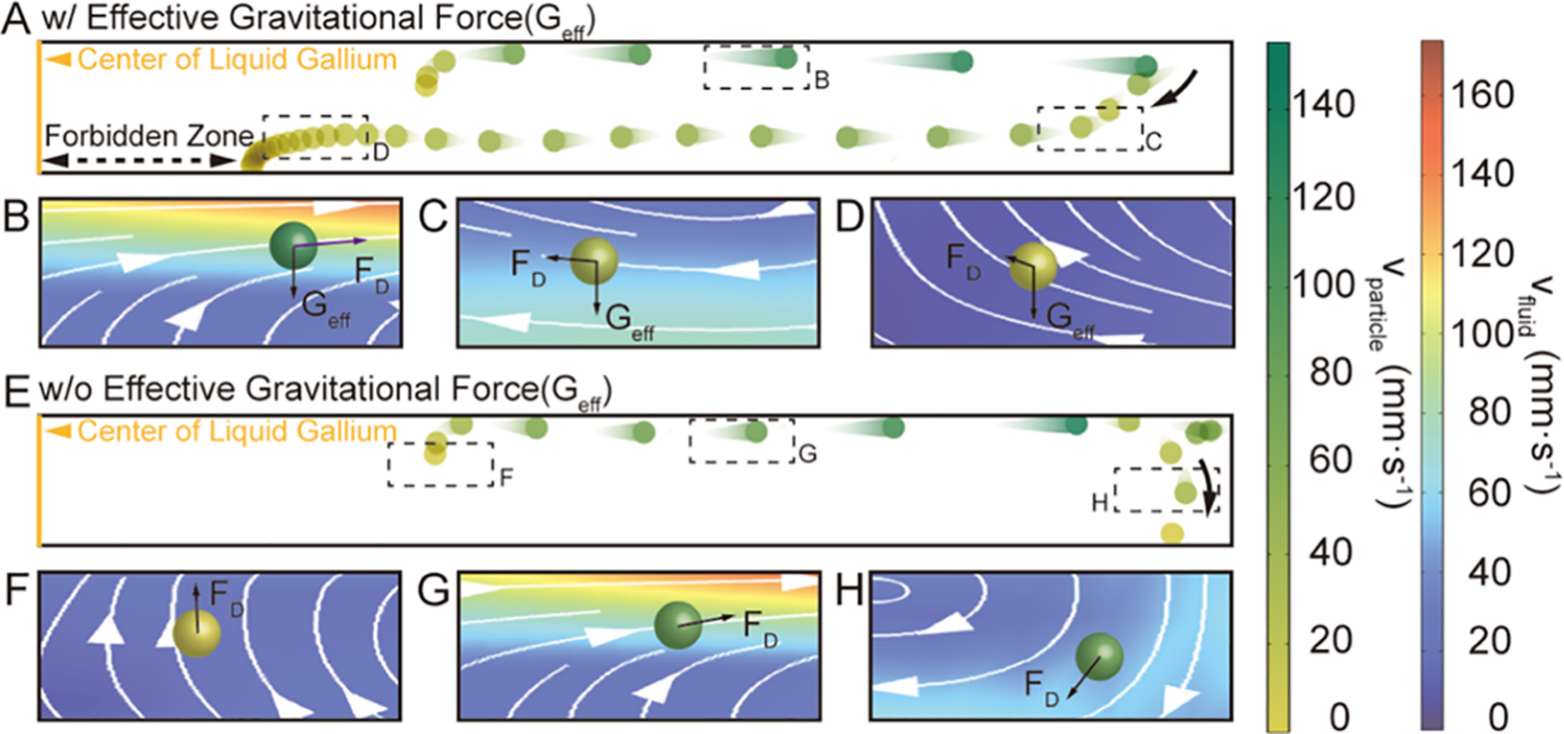
Mechanism for the formation of ring-shaped particle patterns in liquid-gallium under the LG_14_ laser. (A) Trajectory of a representative particle with effective gravitational force. The particle starts near the top surface and settles at the liquid–solid interface (bottom surface). For simplification, only half of the liquid-gallium fluid flow is shown, where the center of liquid-gallium is marked by a solid yellow line. The width of the forbidden zone is marked by a double-headed dashed black arrow. Panels (B, C, and D) highlight the forces exerted on the particle when it: (B) moves along the top surface of liquid-gallium, (C) moves toward the liquid–solid interface, and (D) before it settles at the liquid–solid interface. The direction of the drag forces (*F*_*D*_) and the effective gravitational force (*G*_*eff*_) are shown on the particle (solid black arrows). The background of the fluid velocity with superimposed streamlines illustrates the fluid dynamics of liquid-gallium in the magnified regions. (E) Trajectory of a representative particle without effective gravitational force. The particle starts near the top surface and settles near the periphery of the liquid-gallium boundary. Panels (F, G, and H) highlight the forces exerted on the particle when it: (F) initially is released into the fluid, (G) moves along the top surface of liquid-gallium, and (H) before it settles around the periphery of the liquid-gallium boundary. The absolute values of the fluid velocity (red-to-blue gradient) and particle velocity (green-to-yellow gradient) are indicated by the color bars on the right side of the figure.

The motion of a representative particle moving through the fluid can be seen in Figure 4A with magnified snapshots of this motion with the interacting *G*_eff_ and *F*_D_ forces superimposed in Figure 4B–D. In Figure 4A, the particle picks up drag forces near the surface due to the Marangoni flow. However, before it reaches the sidewalls (Figure 4B), it moves downward due to the gravitational force. The particle then continues to be guided by the convective flow of the fluid while slowly nearing the bottom of the liquid–solid interface (Figure 4C). The velocity of the fluid approaches zero near the boundary of the forbidden zone because the sum of fluid flow directions cancels each other out (Figure 4D). Hence, when particles approach this boundary, the gravitational force on the particle dominates its trajectory. As a result, the particle slowly settled around the periphery of the “forbidden zone,” forming the well-defined ring-shaped pattern seen in Figure 3I.

To further elucidate the role of the gravitational force on the particle pattern formation, a comparative study on the motion of the particles without effective gravitational forces (*G*_eff_ = 0) was performed. As seen in Figure 4E, when a particle initially picked up drag forces due to the Marangoni flow (Figure 4F), it continued to be guided by the fluid flow near the surface until it nears the sidewalls of the liquid-gallium (Figure 4G). There, the particle has a much higher probability to strike the sidewall because of the absence of gravitational forces. Due to the boundary conditions set in this model, this particle will ultimately bounce off the sidewall back into the fluid (See the Methods Section). The particle then continues to be guided by the recirculating convective flow pattern of the fluid (Figure 4H), finally settling at the liquid–solid interface around the sidewall boundaries. Moreover, the influence of the sidewall boundary conditions on the particle motion and particle pattern formation at the liquid–solid interface was also investigated. Despite setting the sticking coefficient of the sidewalls to unity (i.e., all particles that strike the sidewall will stick), remanence of a ring-shaped particle pattern was still observed for particles with diameter of 20 μm (Figure S1A). More importantly, when the particle diameter was reduced to 5 μm, less particles struck the sidewalls, giving rise to a more well-defined ring-shaped pattern (Figure S1B). This was because the probability of impacting the sidewall was reduced with decreasing particle size (more information in Supporting Information—Section IV). Therefore, these results clearly demonstrate that the gravitational force on the particles reduces the particle–sidewall interaction, promoting the formation of a ring-shaped pattern assembly, where the degree of order for the pattern can be further improved through careful tuning of the parameters of the laser itself.

### Tunable Laser Parameters for Particle Assembly

**Figure 5:**
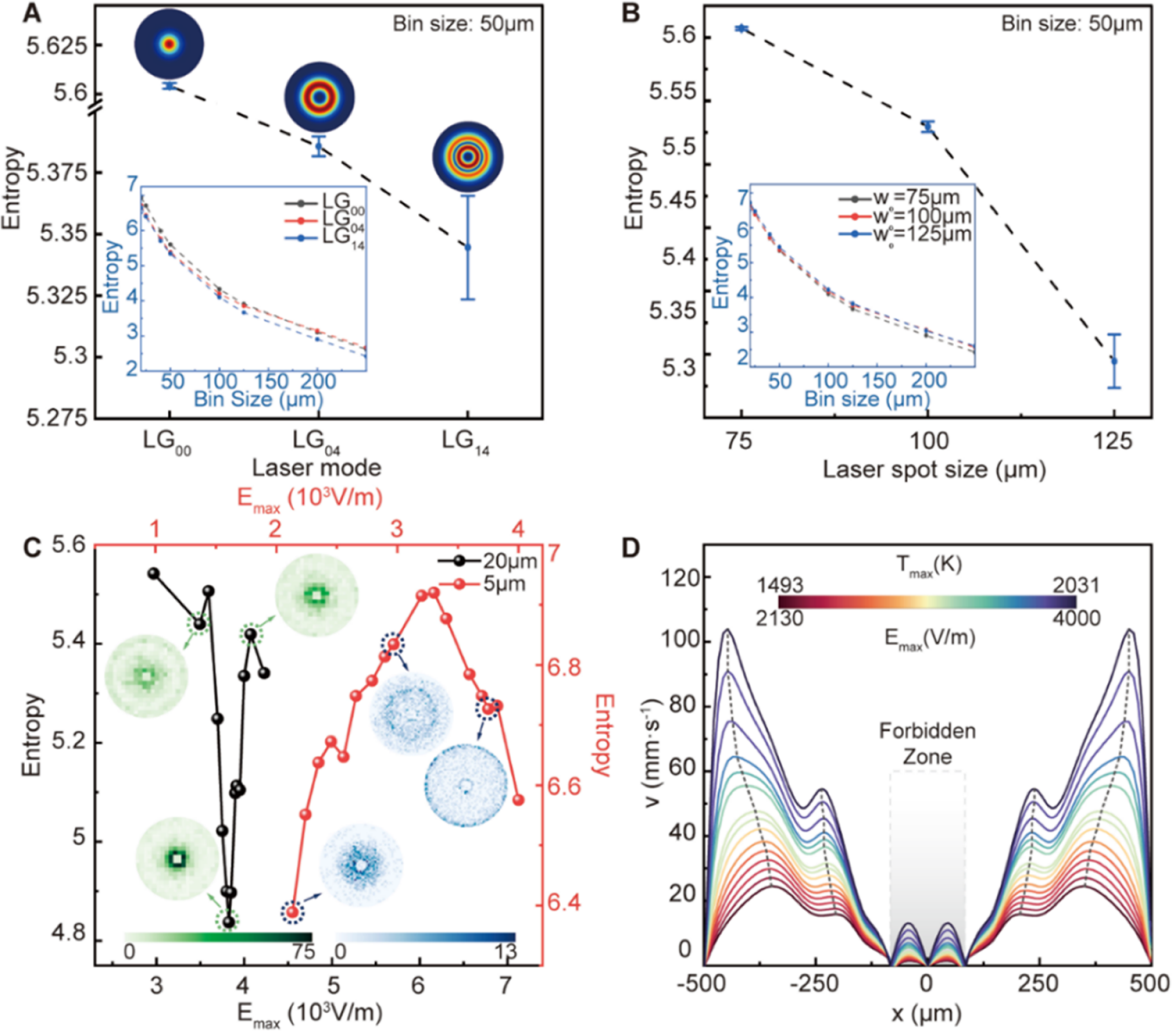
Summary of the tuning knobs of LG_pl_ lasers to obtain a ring-shaped pattern with different degrees of order. (A) Impact of LG_pl_ lasers: entropy of particle patterns (*d*_p_ = 20 μm) under different LG_pl_ lasers (LG_00_, LG_04_, and LG_14_, w_0_ = 125 μm, and *E*_max_ = 4000 V/m). Bin size for the calculation of entropy is 50 μm. The entropy as a function of the bin sizes is also shown in the inset. (B) Impact of LG_pl_ laser spot size: entropy of particle patterns (*d*_p_ = 20 μm) under LG_14_ laser (*E*_max_ = 4000 V/m) with a spot size of 75, 100, and 125 μm. Bin size for the calculation of entropy is 50 μm. The entropy values at different bin sizes are also shown in the inset. (C) Impact of the maximum intensity value of the electric field: entropy of particle patterns under different *E*_max_ values (LG_14_ laser and *w*_0_ = 125 μm). The bin size for the calculation of entropy is 50 for 20 μm diameter particles (solid black line), and the bin size is 20 for 5 μm diameter particles (solid red line). Corresponding density distribution maps for selected data points (dashed circles in panel (C)) are shown. The count of particles in each square grid is indicated by the color bar at the bottom (green-to-white gradient for 20 μm diameter particles and blue-to-white gradient for 5 μm diameter particles). (D) Fluid velocity profile along the solid–liquid interface under *E*_max_ values. The velocity peaks are marked by dashed black lines. The region of the forbidden zone is highlighted in a shaded gray region. *E*_max_ and temperature maxima (*T*_max_) in the liquid-gallium are indicated by the top color bar.

Our results showed that LG_pl_ lasers can be effectively used to engineer the temperature and surface tension of liquid metals to create unique Marangoni flow patterns in the fluid. Although only a ring-shaped pattern assembly was demonstrated in this study, the use of non-Gaussian lasers can more broadly be a simple yet powerful approach to realize hierarchical assembly of particles and other small-scale solutes from liquid metals through appropriate scaling and laser parameter tuning. Therefore, in Figure 5A–D, we summarize what are deemed the most important and tunable parameters of LG_pl_ lasers to design ring-shaped particle assemblies with varying degrees of order. In Figure 5A, the influence of the LG_pl_ lasers on the entropy of the ring-shaped pattern is highlighted. These results show that regardless of the bin size applied to calculate the particle density maps per LG_pl_ laser (Figure 5A, inset), the LG_14_ laser led to particle assemblies with the lowest entropy values when compared to the LG_00_ and LG_04_ lasers. Also, as evident in Figure 5B, enlarging the *w*_o_ of the LG_14_ laser mode was beneficial in improving the degree of order in the ring-shaped pattern assembly independent of the bin size used to calculate the entropy.

Another important parameter that can be controlled in the laser is the maximum intensity value of the electric field (*E*_max_). This allows one to tune the electromagnetic heating of the surface of liquid-gallium. This is highlighted in Figure 5C for both 20 μm (solid black line) and 5 μm (solid red line) particle sizes that assemble out of the liquid-gallium using a LG_14_ laser. The resulting particle density maps that are shown as insets in Figure 5C contained the same number of particles for each electric field condition. For the case of the 20 μm particle size, the entropy values were larger at lower values of the electric field. This was due to the reduced velocity and temperature of the fluid and therefore drag forces on these 20 μm particles. Interestingly, when increasing the electric field, the entropy values of the ring-shaped particle assembly first decreased to a minimum and then rapidly increased. The reduction in entropy at this minimum (19.26% from randomly distributed particles, see the Supporting Information—Section III) stood for the highest degree of order in the ring-shaped particle assembly seen for 20 μm particles using a LG_14_ laser. As evident in the particle density map at *E*_max_ = 3825 V/m (Figure 5C, green map inset), 70.1% of the particles gathered around the “forbidden zone,” which led to this high degree of order (for more detailed particle maps, see Figure S2). No matter the bin size used for the calculation of entropy, the highest degree of order in the ring-shaped particle assembly was always achieved at *E*_max_ = 3825 V/m (Figure S3A). Furthermore, with the smaller 5 μm particle size (red line, Figure 5C), a minimum entropy value in the ring-shaped pattern was achieved at even lower values of electric field when compared to the 20 μm particle (*E*_max_ = 2130 V/m, Figures 5C and S3B). It is expected that a further reduction in the electric field for the 5 μm particle size seen in Figure 5C would further reduce the entropy value (for more detailed particle maps, see Figure S4). This is because the competition between the drag and gravitational forces on the particles, as seen in Figure 4, ultimately dictates the degree of order of the ring-shaped pattern. Since the ratio between the drag forces and the gravitational force is proportional to *d*_P_^–2^, this ratio will increase significantly as the particle size is reduced. Therefore, to improve the entropy of patterns produced with smaller particle sizes, the magnitude of this drag force must be reduced. This can be simply done by lowering the electric field intensity, as corroborated in Figure 5C when comparing this effect on both the 20 and 5 μm particle sizes. To further investigate the influence of the drag coefficient on particle assembly, a comparative study of the drag coefficient derived from the Schiller–Naumann model was performed. For both the Stokes and Schiller–Naumann models, a ring-shaped particle pattern was observed, and the trend of entropy change with the electric field intensity was consistent. The only difference between the two models lies in the *E*_max_ corresponding to the entropy minima, which was higher in the case of the Schiller–Naumann model (*E*_max_ = 3950 V/m, Figure S5) compared to Stokes law (*E*_max_ = 3825 V/m) for 20-μm particles.

Moreover, it is not only possible to control the extent of the forbidden zone through careful choice of the radial and azimuthal index numbers of the LG_pl_ laser, but it is also possible to control the particle dispersion width of the ring-shaped assembly by tuning the velocity of the fluid with the electric field of the laser. Here, the particle dispersion width is qualitatively defined as the radial distance from the boundary of the forbidden zone to the boundary of the liquid-gallium domain wall. The velocity profiles of the fluid as a function of the LG_14_ laser electric field intensity at the liquid–solid interface are shown in Figure 5D. At the boundary of the forbidden zone (Figure 5D, shaded gray region), the velocity of the fluid dropped to zero. Outside of the forbidden zone, undulated fluid velocity profiles can be seen. As the electric field intensity of the laser increases, the absolute value for the fluid velocity also increases. This also shifts the fluid velocity maxima away from the center of liquid-gallium (Figure 5D, dashed black lines). In this case, the drag forces on the particles would also increase with the electric field, making them harder to assemble around the boundary of the forbidden zone. Consequently, particles would be able to assemble out of the liquid-gallium before reaching the boundary of the forbidden zone. This would result in a larger dispersion width in the ring-shaped particle assembly. However, if the fluid velocity becomes too high, the drag forces would dominate the motion of the particles, where particles will prefer to circulate within the convective flow pattern of the fluid at longer time scales, making them more likely to strike the sidewalls. Under this case, particles will gather around the periphery of the liquid-gallium domain wall (Figure S4H). Therefore, to obtain a tighter ring dispersion width, the intensity of the electric field should be kept at a moderate value to maintain a moderate ratio between the drag and gravitational forces, as seen in Figure 4A.

## Conclusions

In summary, light-induced surface tension gradients have proven useful in not only controlling the fluid flow of opaque liquids but also directing the trajectory of dispersed particles within the bulk of liquid gallium, which is commonly dictated by Brownian forces. Overcoming Brownian is imperative to realize the controllable movement of solutes within the bulk of liquids and, thus, to achieve a predictable spatial assembly with a high degree of order, complementary to what has been realized in particle assembly out of ferrofluids using magnetic fields.^43−45^ Our results highlight that assemblies with different degrees of order can be formed by engineering Marangoni flow in liquid-gallium using LG_pl_ lasers, which represent a tunable approach to design particle assemblies out of liquid metals. We found that the synergy between the drag forces on the particle from the convective flow of the fluid and the effective gravitational force is responsible for limiting the influence of Brownian forces on the particle’s motion in the fluid, therefore resulting in the formation of unique ring-shaped particle assemblies at the liquid–solid interface with a high degree of tunability. Careful control over the parameters of the LG_pl_ laser (i.e., laser mode, spot size *w*_*o*_, and intensity of the electric field *E*_max_, etc.) can tune the temperature and fluid dynamics of the liquid-gallium as well as the balance of forces on the particle, which in turns can tune the structure of the ring-shaped particle pattern between the coffee-ring and reverse coffee-ring effects. This is a result of prominent central vortices within the convective flow of the bulk liquid that creates a forbidden zone of assembly at the liquid–solid interface. A striking example of this is when 70.1% of randomly dispersed tungsten particles in liquid-gallium under the LG_14_ laser mode (*d*_p_ = 20 μm, *w*_o_ = 125 μm, and *E*_max_ = 3825 V/m) gathered around the periphery of the forbidden zone at the liquid–solid interface, forming a 100 μm wide ring-shaped particle assembly that was smaller than the beam size of the laser itself.

It has been shown that electric fields can be used to modulate the wetting behavior of many liquid metals.^46−49^ Although this study has focused on the interaction of lasers with liquid-gallium, this approach can be extended to other liquid metals, such as mercury (Hg), indium (In), tin (Sn), etc., or eutectic metal alloy systems, such as Galinstan, field’s metal, etc. The variety of liquid metals that are available with different melting points, viscosities, solubilities, and reactivities will further expand the design space for creating complex material designs from a variety of elements and particle structures dissolved and/or dispersed in these fluids across different length scales from the bottom-up. Additionally, the use of a common laser as a heating source to develop Marangoni flow in liquid metals opens the possibility of achieving rapid spatial switching in the design of the temperature gradients on the liquid metal and therefore the surface tension gradients. The modularity of this fabrication process could potentially allow for an on-demand spatial control over the precipitation of solutes out of the liquid metal, such as that seen in crystal growth processes of nanostructures, like the VLS mechanism.^50,51^ This can be done by altering the input of the laser beam shape and profile of the electric field using spatial light modulators and other appropriate optical components.

## Methods

### Framework for Coupling the Marangoni Effect and Fluid Flow

In this study, liquid-gallium was placed with cylindrical boundary conditions and in a nonoxidizing environment to simplify the model and avoid the influence of surface curvature on surface tension at the two-phase interface. The steady-state Marangoni flow pattern in liquid-gallium induced by the Laguerre-Gaussian laser mode was simulated by coupling the laminar flow, heat transfer in the fluid, and electromagnetic wave using a combination of finite element method solver packages (COMSOL Multiphysics). Herein, the laminar flow in liquid-gallium was governed by the Navier–Stokes equation
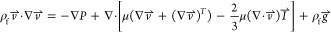
where ρ_*f*_ is the density of liquid-gallium, *v⃗* is the fluid velocity, *P* is the pressure in liquid-gallium, μ is the viscosity of liquid-gallium, and *g⃗* is the gravitational acceleration constant. The heat transfer in the fluid was coupled to the movement of the fluid by combing *v⃗* and *P*. The heat transfer in liquid-gallium was described with the conservation of energy given by
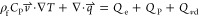

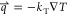
where *C*_P_ is the heat capacity and *k*_T_ is the thermal conductivity of liquid-gallium, respectively. Moreover, the temperature gradient that develops on the surface of liquid-gallium induces sheer stresses at the gas–liquid interface giving rise to Marangoni flow
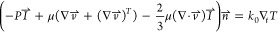
where *v⃗* is the velocity of the fluid and *k*_0_ is the temperature coefficient of surface tension of the fluid. In the case of liquid-gallium, this *k*_0_ is negative.

### Laser Heating Setup and Conditions

The major heating source was by electromagnetic heating, which is given by
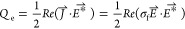
where σ_f_ is the electrical conductivity and *J* is the current density of the liquid-gallium. In our models, the electromagnetic sources are three different Laguerre-Gaussian laser modes (i.e., LG_00_, LG_04_, and LG_14_ laser modes, propagating in the *z*-direction), which are described by
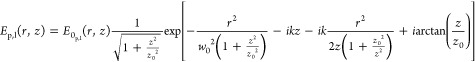
where *z*_0_ is the Rayleigh range of the laser and *E*_0_p,l__(*r*,*z*) is the amplitude for the electric field distribution of the LG_pl_ laser mode.

For LG_00_ laser mode
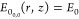
For LG_04_ laser mode
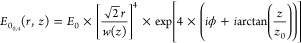
For LG_14_ laser mode
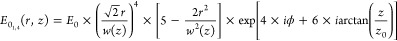

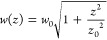

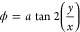
Furthermore, the work done by pressure change in liquid-gallium (*Q*_P_) is given by
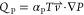
where α_P_ is the coefficient of thermal expansion of liquid-gallium. The viscous dissipation in liquid-gallium (*Q*_vd_) is given by
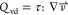
where τ is the viscous tensor. At the boundary of liquid-gallium, radiation heating from the liquid-gallium to ambient (air) was taken into consideration. The net outward radiative flux (*q*_*r,*net_) is given by
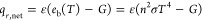

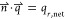
where ε and *n* are the emissivity and refractive index of liquid-gallium at 645 nm, respectively. σ is the Stefan–Boltzmann constant, and *G* is the total incoming radiative flux. *n⃗* is the unit normal vector.

### Particle Trajectory Simulation Setup

Particle trajectories were simulated under steady-state fluid flow. A random distribution of 2000 neutrally charged tungsten particles was consistently released in the liquid-gallium for a fair comparison. The particle trajectory was simulated based on Newton’s second law
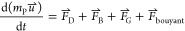
where  is the drag force from the fluid flow,  is the Brownian force,  is the gravitational force, and  is the buoyant force. To guarantee the accuracy of the simulated particle trajectories, the time step was set to the characteristic time of the particle (τ_P_), which is given by
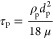
The characteristic time for the 20 and 5 μm tungsten particles was (6.72 × 10^–4^ s and 4.2 × 10^–5^) s, respectively. Gravitational, buoyant, Brownian, and drag forces were applied to the particles. For the boundary conditions, all particles bounced back elastically, except at the liquid–solid interface where assembly of particles can occur (bottom side of liquid-gallium). For the liquid–solid interface, a sticking probability of 50% was included in our models; otherwise, the particle would be able to bounce back into the fluid elastically. To simplify the calculations, the heat transfer between particles and liquid-gallium was neglected due to the low concentration of particles in the fluid (<10^–15^ mol/L). Also, the particles are not interacting with each other. More details on the model settings are included in the Supporting Information.

## Data Availability

All data needed to evaluate the conclusions in the paper are present in the paper and/or the Supporting Information.

## Supporting Information Available

Additional information, including details of physics setting for the particle trajectory simulation, calculation of mean-squared displacement for particle trajectory, entropy calculation of the particle assemblies, probability of the particle–sidewall interactions, and particle patterns under different maximum intensity values of the electric field (PDF)
