# Supplementary material for: Light-Induced Surface Tension Gradients for Hierarchical Assembly of Particles from Liquid Metals
Source: ACS Appl Mater Interfaces. 2023 Feb 2;15(7):10182–92. doi: 10.1021/acsami.2c20116 (PMC9951180; doi:10.1021/acsami.2c20116)
Supplement: Supplementary file 1 — am2c20116_si_001.pdf [file am2c20116_si_001.pdf]

Supporting information

Light Induced Surface Tension Gradients for Hierarchical Assembly of Particles from Liquid Metals

Jiayun Liang[1], Zakaria Y. Al Balushi[1,2*]

[1]Department of Materials Science and Engineering, University of California, Berkeley; Berkeley, CA 94720, USA.

[2]Materials Sciences Division, Lawrence Berkeley National Laboratory, Berkeley, CA 94720, USA.

*Corresponding author, e-mail: albalushi@berkeley.edu

This PDF file includes:

Supporting Text

- Sec. I: Physics setting for the particle trajectory simulation
- Sec. II: Mean-squared displacement for the particle trajectory
- Sec. III: Entropy calculation of the particle assemblies
- Sec. IV: Probability of particle-sidewall interaction

Supporting Tables

- Table S1: Summary of elements that do not form compounds with liquid gallium stable above the energy hull cutoff
- Table S2: Parameters for finite element model

Supporting Figures

- Figure S1. XY cross-section of the particle assembly with the sticking coefficient of the sidewalls set to unity (LG_{14} laser mode and $w_0 = 125\ \mu m$)
- Figure S2. Particle patterns under different maximum intensity value of the electric field (E_{max}) for particles with a diameter of $20\ \mu m$ (LG_{14} laser mode and $w_0 = 125\ \mu m$)
- Figure S3. Entropy of the particle pattern under different maximum intensity value of the electric field (E_{max}) calculated with different bin sizes (LG_{14} laser mode and $w_0 = 125\ \mu m$)
- Figure S4. Particle patterns under different maximum intensity value of the electric field (E_{max}) for particles with a diameter of 5 μm (LG_{14} laser mode and $w_0 = 125\ \mu m$)
- Figure S5. Entropy of particle patterns under different E_{max} values (LG_{14} laser mode and $w_0 = 125\ \mu m$) with drag coefficient from Schiller-Naumann model.
- Figure S6. Impacts of mesh element number on the simulation result accuracy
- Figure S7. Statistical analysis of the entropy calculations

SUPPORTING TEXT

Sec. I: Physics setting for the particle trajectory simulation

The steady-state Marangoni flow pattern in liquid gallium induced by the Laguerre-Gaussian laser mode was simulated by coupling the laminar flow, the heat transfer in the fluid and the electromagnetic wave using a finite element method solver COMSOL Multiphysics®. The size of liquid gallium was $1000\ \mu m$ in diameter and $50\ \mu m$ in height. To guarantee the accuracy of the simulation results, an extremely fine and symmetric mesh containing 173,888 triangle elements was built. The solved results (e.g., electric field profile, temperature profile, etc.) stabilized as the mesh element number is greater than 145,800. (Figure S6)

The particle trajectory was simulated based on Newton's second law:

$$\frac{d(m_P\vec{u})}{dt} = \overrightarrow{F_D} + \overrightarrow{F_B} + \overrightarrow{F_G} + \overrightarrow{F_{Bouyant}} \tag{1}$$

where $\overrightarrow{F_D}$ is the drag force from the fluid flow, $\overrightarrow{F_B}$ is the Brownian force, $\overrightarrow{F_G}$ is the gravitational force, and $\overrightarrow{F_{Bouyant}}$ is the buoyant force. Here, the drag force from the fluid flow ($\overrightarrow{F_D}$) is given by:

$$\overrightarrow{F_D} = \frac{m_P(\vec{u} - \vec{v})}{\tau_P} \tag{2}$$

where $\vec{u}$ is the velocity of particle, $\vec{v}$ is the velocity of fluid flow, and τ_P is the characteristic time for the particle. The amplitude of the Brownian force ($\overrightarrow{F_B}$) is given by:

$$\overrightarrow{F_B} = \zeta\sqrt{\frac{6\pi k_B \mu T d_p}{\tau_P}} \tag{3}$$

where k_B is the Boltzmann constant and ζ is a vector of independent, normally distributed random numbers with zero mean and unit standard variation as indicated in COMSOL. For the sticking probability of the particles at the liquid-solid interface was set to 50% (bottom side of liquid gallium). When particle stick on the boundary:

$$\vec{u} = \vec{0} \tag{4}$$

otherwise, the particle would bounce elastically back into the fluid:

$$\vec{u} = \overrightarrow{u_c} - 2(\vec{n} \cdot \overrightarrow{u_c})\vec{n} \tag{5}$$

where $\overrightarrow{u_c}$ is the particle velocity when the particle approaches the boundary of the liquid gallium domain. At all other boundaries of liquid gallium, the particles would bounce back elastically.

Sec. II: Mean-squared displacement for the particle trajectory

The mean-squared displacement ($MSD\ (\tau_P)$) for the particle trajectory is

given by:

$$MSD(\tau_P) =< [\vec{r}(t+\tau_P) - \vec{r}(t)]^2 > \quad (6)$$

where $\vec{r}$ is the position of the particle at time t and τ_P is the time step for the simulation. For all the datasets presented in Figure 2 of the main text, the time step was set as the characteristic time of the particle, i.e., for $20\ \mu m$ tungsten particles, τ_P was $6.72 \times 10^{-4}\ s$.

Sec. III: Entropy calculation of the particle assemblies

In all results, the number of particles that were released into liquid gallium was 2000. We then used entropy to quantify the degree of order of the particle pattern assembly at the liquid-solid interface. To calculate the entropy for different particle assemblies, we equally divide the particle pattern into $(\frac{1000}{n})^2$ square lattices (i.e., "bin") of size n and counted the number of particles in each bin. Here, the entropy for the patterns is defined as:

$$S = -\sum_{i=1}^{\left(\frac{1000}{n}\right)^2} \frac{N_i}{2000} ln \frac{N_i}{2000} \quad (7)$$

where N_i is the number of particles in the ith bin.

To test the reproducibility of our calculations, we repeated each simulation 10 times and compared the entropy for the particle assembly obtained in different runs. A series of bin sizes were utilized for entropy calculation to avoid the impact of the bin size on the entropy results. As shown in Figure S7, no matter the size of the bin utilized, the relative entropy difference was less than 0.8% and the mean squared error was less than 0.6%, supporting the reproducibility of our calculations.

In addition, to quantify the enhancement of the degree of the order, we set the entropy for a completely random particle assembly composed of 2000 tungsten particles as the reference value (S_{ref}). In a completely random particle assembly, the possibility for a particle to assemble at different positions is the same. Therefore, with a $50\ \mu m$ bin size, the entropy for a completely random particle assembly is given by:

$$S_{ref} = -\sum_{i=1}^{400} \frac{5}{2000} ln \frac{5}{2000} = 5.9915 \quad (8)$$

Therefore, the reduction in entropy (ΔS) is given by:

$$\Delta S = \frac{S_{ref} - S_{particle\ assembly}}{S_{ref}} \quad (9)$$

where $S_{particle\ assembly}$ is the entropy for the specific particle assembly of interest. S_{ref} and $S_{particle\ assembly}$ are calculated with the same bin size.

Sec. IV: Probability of particle-sidewall interaction

A particle can only interact with the sidewall when the distance between the particle and the sidewall is no more than the radius of the particle itself. Based on this assumption, the probability for the particle-sidewall interaction (P_0) with different particle size can be given by:

$$P_0 = \frac{\pi\left({d_f}^2 - \left(d_f - d_p\right)^2\right)}{\pi {d_f}^2} = \frac{2d_f d_p - {d_P}^2}{{d_f}^2} \qquad (10)$$

where d_f is the diameter of the liquid gallium ($1000\,\mu m$ in our model). The probability of the particle-sidewall interaction for $20\,\mu m$ diameter particles is 3.96%, while for $5\,\mu m$ diameter particles, the probability drops down to 0.998%. Therefore, as shown in Figure S1, for $5\,\mu m$ diameter particles, less particles stick to the sidewalls (Figure S1B, sticking probability = unity), compared to the pattern produced from $20\,\mu m$ diameter particles (Figure S1A, sticking probability = unity).

SUPPORTING TABLES

Table S1. Summary of elements that do not form compounds with liquid gallium stable above the energy hull cutoff [1-7]

H	He	Be
B	C	Ne
Al	Si	Ar
Zn	Ge	Kr
Cd	In	Sn
Xe	W	Hg
Tl	Pb	Bi

Table S2. Parameters for finite element model [8-9]

Parameter	Physical Meaning	Value
$\boldsymbol{k_0}$	Temperature coefficient of surface tension for liquid gallium	-6.6*10^{-5} $N/(m * K)$
$\boldsymbol{C_P}$	Heat capacity for liquid gallium	381.072862 $J/(kg * K)$
$\boldsymbol{\rho}$	Density of liquid gallium	5592 kg/m^3
$\boldsymbol{\rho_p}$	Density of tungsten particle	1930 kg/m^3
$\boldsymbol{k_T}$	Thermal conductivity of liquid gallium	8.0844 $W/(m * K)$
$\boldsymbol{\alpha}$	Thermal expansion of liquid gallium	3.0762
$\boldsymbol{\mu}$	Dynamic viscosity of liquid gallium	0.64288 $mPa * s$

$\boldsymbol{\sigma_f}$	Electric conductivity of liquid gallium	7.1×10^6 S/m
$\boldsymbol{n}$	Real part of refractive index for liquid gallium at 645nm	$n = 1.57434 + 6.38 * 10^{-4} * (T - 273.15)$
$\boldsymbol{k}$	Imaginary part of refractive index for liquid gallium at 645nm	$k = 7.48476 + 2.46 * 10^{-3} * (T - 273.15) + 2.18 * 10^{-6} * (T - 273.15)^2$
$\boldsymbol{\varepsilon}$	Emissivity of liquid gallium at 645 nm	$\varepsilon = 0.0771 + 6.852 * 10^{-5} * T$

SUPPORTING FIGURES

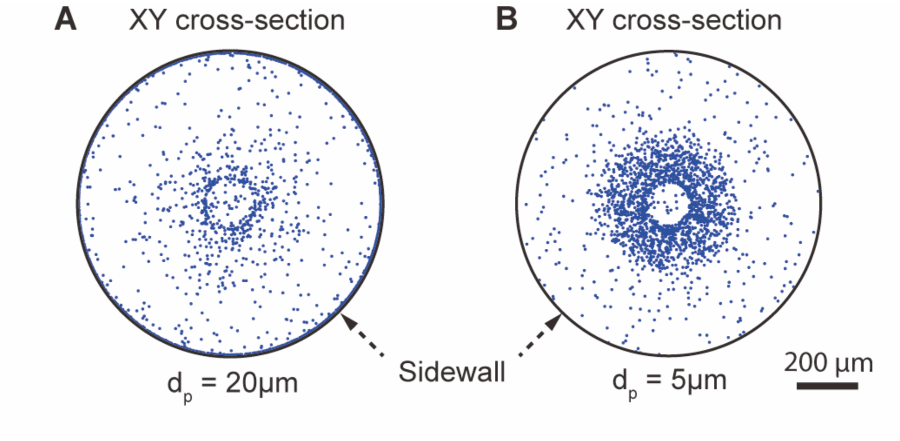

Figure S1. XY cross-section of the particle assembly with the sticking coefficient of the sidewalls set to unity (LG_{14} laser mode and $w_0 = 125\ \mu m$). The diameter of the particles (d_p) are: **(A)** $20\ \mu m$, and **(B)** $5\ \mu m$, respectively. The sidewall is marked with *solid black line* and highlighted with a *dashed black arrow*. Scale bar for the particle pattern is $200\ \mu m$.

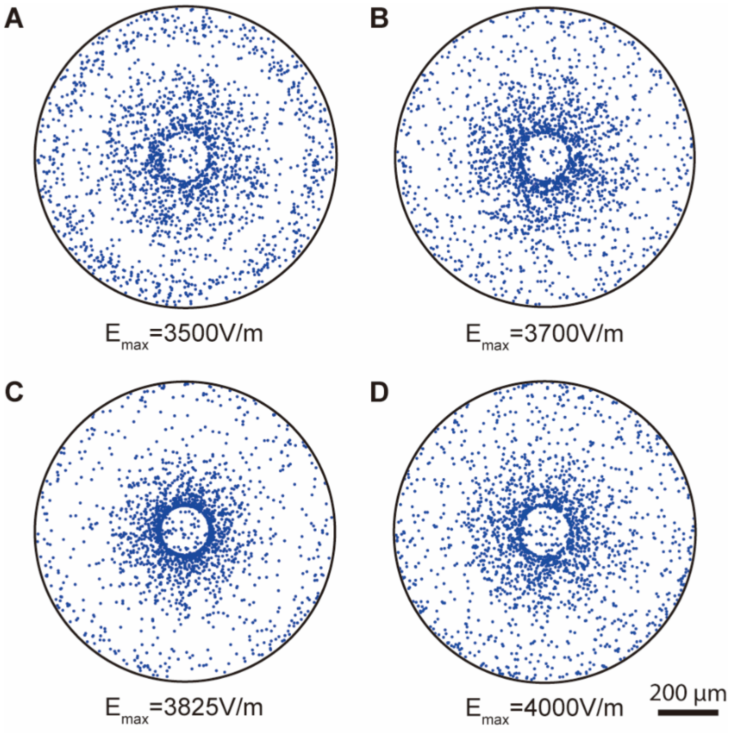

Figure S2. Particle patterns under different maximum intensity value of the electric field ($\boldsymbol{E_{max}}$) for particles with a diameter of $\mathbf{20}\ \boldsymbol{\mu m}$ ($\boldsymbol{LG_{14}}$ laser mode and $\boldsymbol{w_0} = \mathbf{125}\ \boldsymbol{\mu m}$). (A) $E_{max} = 3500\ V/m$, **(B)** $E_{max} = 3700\ V/m$, **(C)** $E_{max} = 3825\ V/m$, and **(D)** $E_{max} = 4000\ V/m$. Scale bar for the particle pattern is $200\ \mu m$.

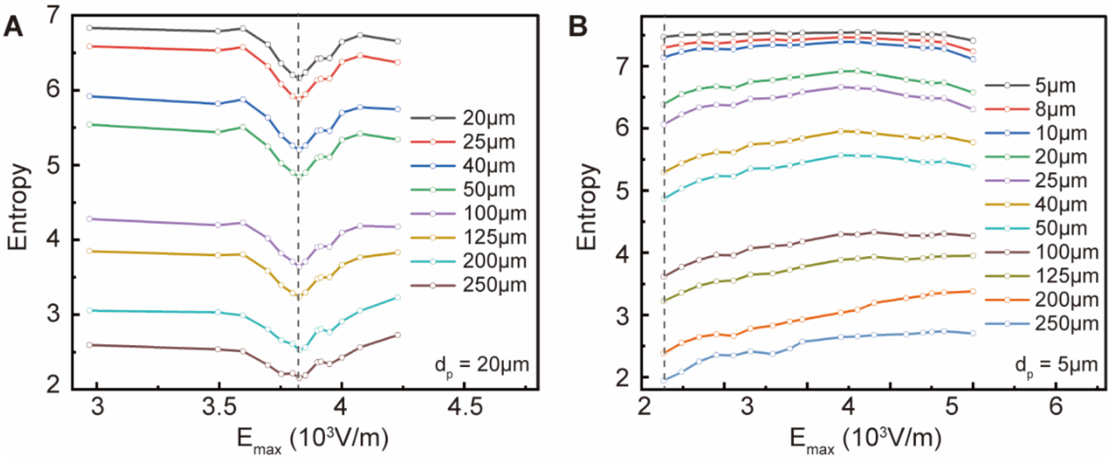

Figure S3. Entropy of the particle pattern under different maximum intensity value of the electric field ($\boldsymbol{E_{max}}$) calculated with different bin sizes ($\boldsymbol{LG_{14}}$ laser mode and $\boldsymbol{w_0} = \mathbf{125}\ \boldsymbol{\mu m}$). The diameter of the particles (d_p) are: **(A)** $20\ \mu m$, and **(B)** $5\ \mu m$, respectively.

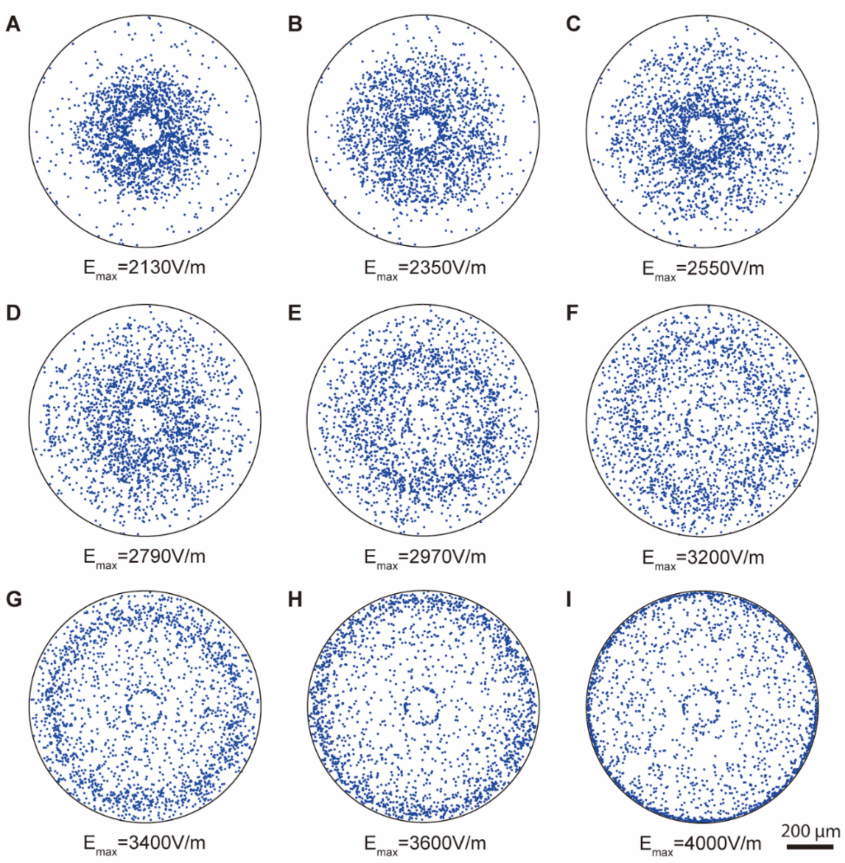

Figure S4. Particle patterns under different maximum intensity value of the electric field ($\boldsymbol{E_{max}}$) for particles with a diameter of 5 $\boldsymbol{\mu m}$ ($\boldsymbol{LG_{14}}$ laser mode and $\boldsymbol{w_0 = 125\ \mu m}$). **(A)** $E_{max} = 3500\ V/m$, **(B)** $E_{max} = 2130\ V/m$, **(C)** $E_{max} = 2350\ V/m$, **(D)** $E_{max} = 2550\ V/m$, **(E)** $E_{max} = 2790\ V/m$, **(F)** $E_{max} = 2970\ V/m$, **(G)** $E_{max} = 3400\ V/m$, **(H)** $E_{max} = 3600\ V/m$, and **(I)** $E_{max} = 4000\ V/m$. Scale bar for the particle pattern is $200\ \mu m$.

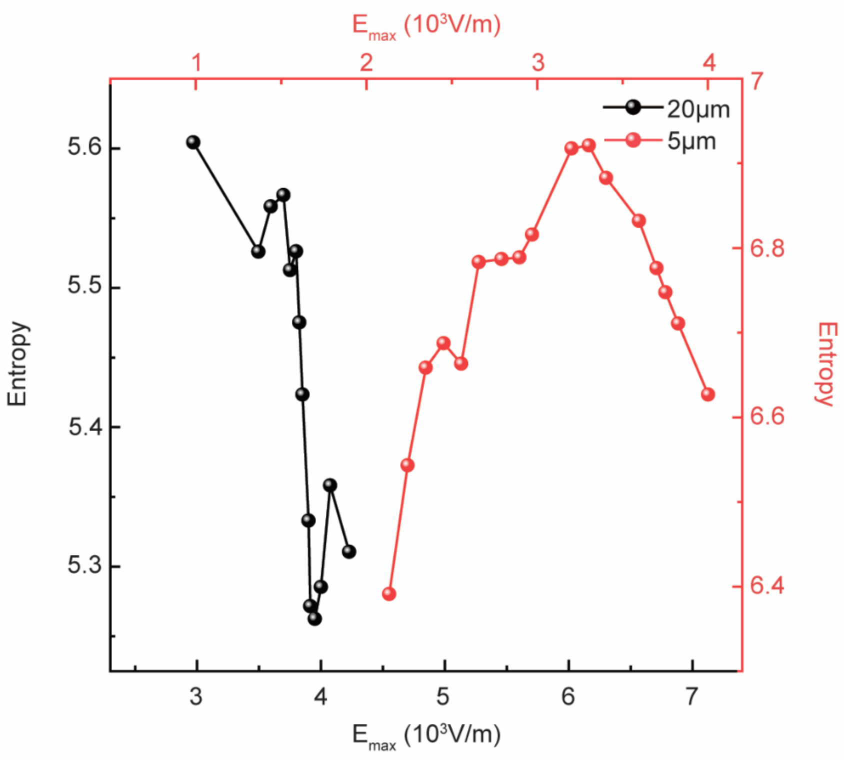

Figure S5. Entropy of particle patterns under different E_{max} values (LG_{14} laser mode and $w_0 = 125\ \mu m$) with drag coefficient from Schiller-Naumann model. The bin size for the calculation of entropy is $50\ \mu m$ for $20\ \mu m$ diameter particles (*solid black line*), and the bin size is $20\ \mu m$ for $5\ \mu m$ diameter particles (*solid red line*).

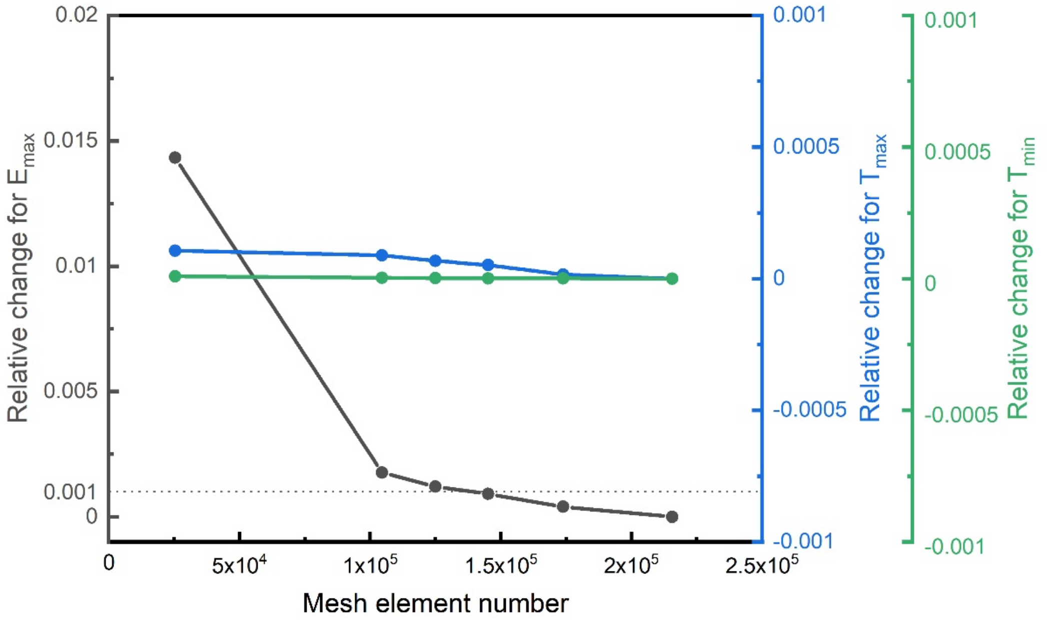

Figure S6. Impacts of mesh element number on the simulation result accuracy. Relative change of: (left y-axis, *black circle*) maximum value of electric field (E_{max}), (right y-axis, *blue circle*) maximum value of temperature in liquid gallium (T_{max}), (left y-axis, *green circle*) minimum value of temperature in liquid gallium (T_{min}).

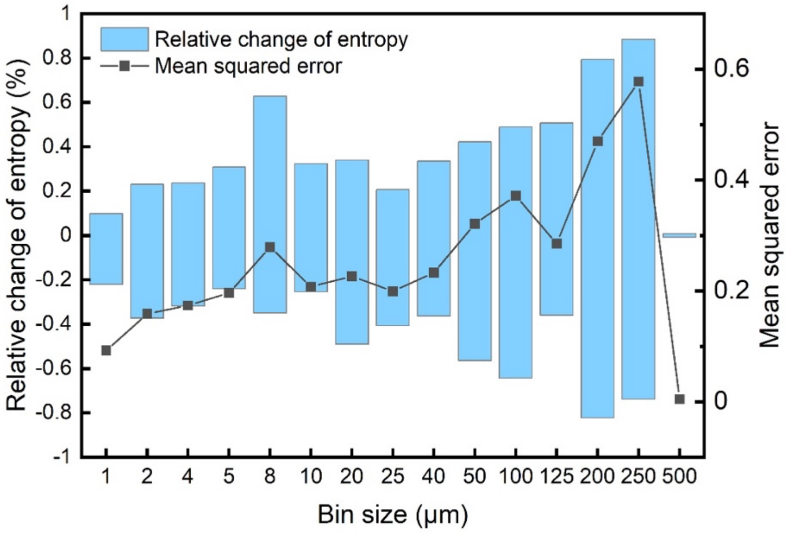

Figure S7. Statistical analysis of the entropy calculations. Relative change of entropy (left y-axis, *blue box*) and mean squared error of entropy (right y-axis, black solid line) for the particle assembly obtained in 10 repeated runs.

SUPPORTING REFERENCES

1. Wald, F.; Stormont, R. W. Investigations on the Constitution of Certain Binary Boron-Metal Systems. *Journal of the Less Common Metals* **1965,** *9*, 423-433.
2. Yatsenko, S.; Sabirzyanov, N.; Yatsenko, A. In *Dissolution Rates and Solubility of Some Metals in Liquid Gallium and Aluminum*, Journal of Physics: Conference Series, IOP Publishing: **2008**; p 062032.
3. Hiura, H.; Lee, M. V.; Tyurnina, A. V.; Tsukagoshi, K. Liquid Phase Growth of Graphene on Silicon Carbide. *Carbon* **2012,** *50*, 5076-5084.
4. Lee, M. V.; Hiura, H.; Kuramochi, H.; Tsukagoshi, K. Concerted Chemical-Mechanical Reaction in Catalyzed Growth of Confined Graphene Layers into Hexagonal Disks. *The Journal of Physical Chemistry C* **2012,** *116*, 9106-9113.
5. Lee, M. V.; Hiura, H.; Tyurnina, A. V.; Tsukagoshi, K. Controllable Gallium Melt-Assisted Interfacial Graphene Growth on Silicon Carbide. *Diamond and related materials* **2012,** *24*, 34-38.
6. Jain, A.; Ong, S. P.; Hautier, G.; Chen, W.; Richards, W. D.; Dacek, S.; Cholia, S.; Gunter, D.; Skinner, D.; Ceder, G. Commentary: The Materials Project: A Materials Genome Approach to Accelerating Materials Innovation. *APL materials* **2013,** *1*, 011002.
7. Li, X.; Cheng, K.; Yuan, X.; Zhao, D.; Xin, J.; Wang, W.; Zhang, C.; Du, Y. Thermodynamic Assessment of the Ga–X (X= B, Ca, Sr, Ba) Systems Supported by First-Principles Calculations. *Calphad* **2013,** *43*, 52-60.
8. R. L. Montgomery, P. C. S., D. W. Ball, J. L. Margrave Thermodynamic Properties by Levitation Calorimetryn V. High-Temperature Heat Content of Liquid Gallium. *International Journal of Thermophysics* **1984,** *5*, 161-175.
9. Teshev, R. S.; Shebzukhov, A. Electronic Characteristics and Dispersion of Optical Constants of Liquid Gallium in the 0.4-2.5-μm Spectral Region. *Optics and Spectroscopy* **1988,** *65*, 693-695.
